# Crystal structure and Hirshfeld-surface analysis of (benzene­carbo­thio­amide-κ*S*)bromido­bis­(tri­phenylphosphane-κ*P*)silver(I)

**DOI:** 10.1107/S2056989016009518

**Published:** 2016-06-21

**Authors:** Wattana Ruangwut, Saowanit Saithong, Chaveng Pakawatchai

**Affiliations:** a Department of Chemistry and Center of Excellence for Innovation in Chemistry, Faculty of Science, Prince of Songkla University, Hat Yai, Songkhla 90112, Thailand

**Keywords:** crystal structure, Ag^I^ complex, benzene­carbo­thio­amide, tri­phenyl­phosphane, hydrogen bonding, Hirshfeld surface, supra­molecular inter­action

## Abstract

The title complex, [AgBr(C_7_H_7_NS)(C_18_H_15_P)_2_], was obtained from the reaction of silver(I) bromide with benzene­carbo­thio­amide (C_7_H_7_NS) and tri­phenyl­phosphane (C_18_H_15_P) in the mixed solvent of aceto­nitrile and ethanol. The mononuclear complex exhibits a distorted tetra­hedral coordination geometry about the metal atom, arising from one S atom of a benzene­carbo­thio­amide ligand, two P atoms of two tri­phenyl­phosphane mol­ecules and one bromide ion. An intra­molecular N—H⋯Br hydrogen bond is observed and in the crystal structure, inversion dimers linked by pairs of N—H⋯Br and C—H⋯Br hydrogen bonds are observed. In addition, C—H⋯π inter­actions occur, leading to [101] chains. Hirshfeld-surface analyses are presented and discussed.

## Abstract 2

The mononuclear complex exhibits a distorted tetra­hedral coordination geometry about the metal atom, arising from one S atom of the benzene­carbo­thio­amide ligand, two P atoms of two tri­phenyl­phosphane mol­ecules and one bromide ion. An intra­molecular N—H⋯Br hydrogen bond is observed and in the crystal structure, inversion dimers linked by pairs of N—H⋯Br and C—H⋯Br hydrogen bonds are observed. In addition, C—H⋯π inter­actions occur, leading to [101] chains.

## Chemical context   

Mixed-ligand complexes of Ag^I^-containing phospho­rus and sulfur donor ligands have been studied and published extensively in recent years (Dennehy *et al.*, 2007 ▸; Ruangwut & Pakawatchai, 2014 ▸) because of their potential ability to inhibit bacteria (Isab *et al.*, 2010 ▸; Nawaz *et al.*, 2011 ▸). Tri­phenyl­phosphane and thione ligands, which contain P and S donor atoms, respectively, are capable of forming mixed-ligand silver(I) complexes as mononuclear (Aslanidis *et al.*, 1997 ▸) and dinuclear models (Cox *et al.*, 2000 ▸). In this paper, we report the synthesis and structure of the mixed-ligand complex of silver(I) bromide with tri­phenyl­phosphane and benzene­carbo­thio­amide ligands.
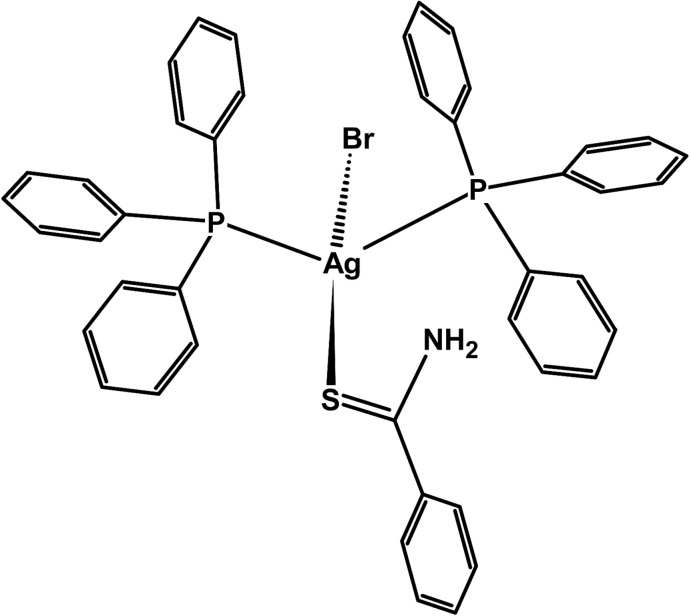


## Structural commentary   

**Figure 1:**
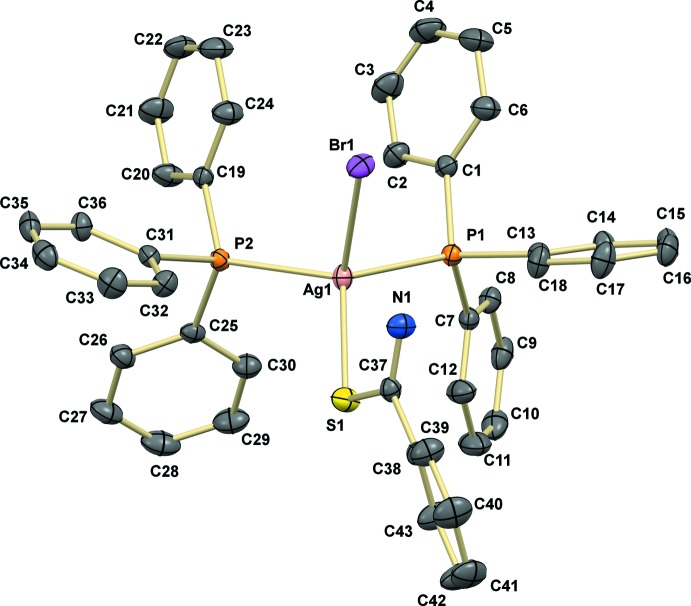
The mol­ecular structure of the title compound, showing 30% probability displacement ellipsoids.

**Figure 2:**
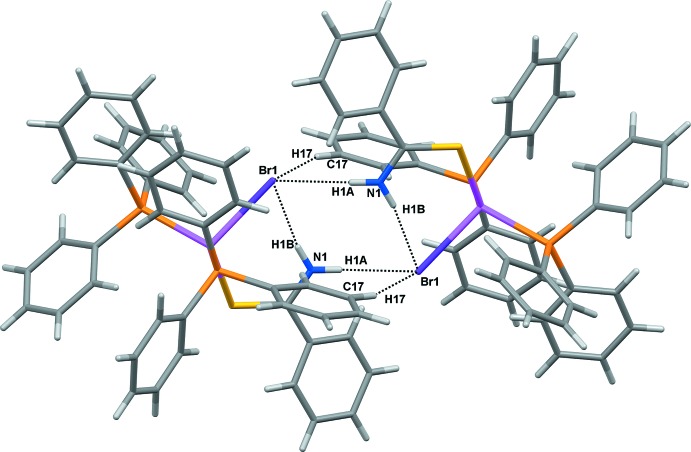
An inversion dimer in the crystal of the title compound linked by two pairs of N—H⋯Br inter­actions, forming 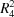
(8) loops, and pairs of C—H⋯Br inter­actions, forming 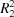
(14) loops.

The monomeric complex of the title compound crystallizes in the monoclinic crystal system, space group *P*2_1_/*n*, and is shown in Fig. 1 ▸. The silver ion is four-coordinated exhibiting a distorted tetra­hedral environment. This deviation can be explained by P1—Ag1—P2 angle which has the highest value of 121.60 (2)° due to the steric hindrance and the repulsion between two bulky tri­phenyl­phosphane mol­ecules. The range of angles around the Ag atom of 97.338 (18)–121.60 (2)° is similar to that observed in the analogous mononuclear silver(I) complex [AgCl(C_7_H_7_NS)(C_18_H_15_P)_2_] previously synthesized by us (Ruangwut & Pakawatchai, 2014 ▸), in which the angles about the metal ion are 97.298 (16)–120.053 (16)°. The Ag—S bond length of 2.6015 (8) Å is slightly longer than in [AgCl(C_7_H_7_NS)(C_18_H_15_P)_2_], 2.5580 (5) Å. The Ag—P bond lengths of 2.4682 (7) and 2.4671 (6) Å for Ag1—P1 and Ag1—P2, respectively, are similar to those of the Ag—P bond lengths in [AgCl(C_7_H_7_NS)(C_18_H_15_P)_2_] [2.4529 (5) and 2.4578 (5) Å], and similar to the Ag—P distances of analogous tetra­hedrally coordinated Ag^I^ complexes such as [Ag(NO_3_)(C_2_H_3_N_3_S)(C_18_H_15_P)_2_] [2.4485 (6) and 2.4493 (6) Å; Wattanakanjana *et al.*, 2014 ▸], [Ag(Htsa)(PPh_3_)_3_] [2.574 (7)–2.611 (6) Å; Nomiya *et al.*, 1998 ▸] and [Ag(PPh_3_)_2_(bzoxtH)]·2NO_3_ [2.480 (1) and 2.514 (2) Å; McFarlane *et al.*, 1998 ▸]. An intra­molecular hydrogen bond N1—H1*B*⋯Br1 [3.413 (3) Å; Table 1 ▸] is found between one of the H atoms from an amine group of the benzene­carbo­thio­amide mol­ecule and the bromide ion, as depicted in Fig. 2 ▸, which also shows the inter-mol­ecular dimeric hydrogen bonds.

## Supra­molecular features   

**Figure 3:**
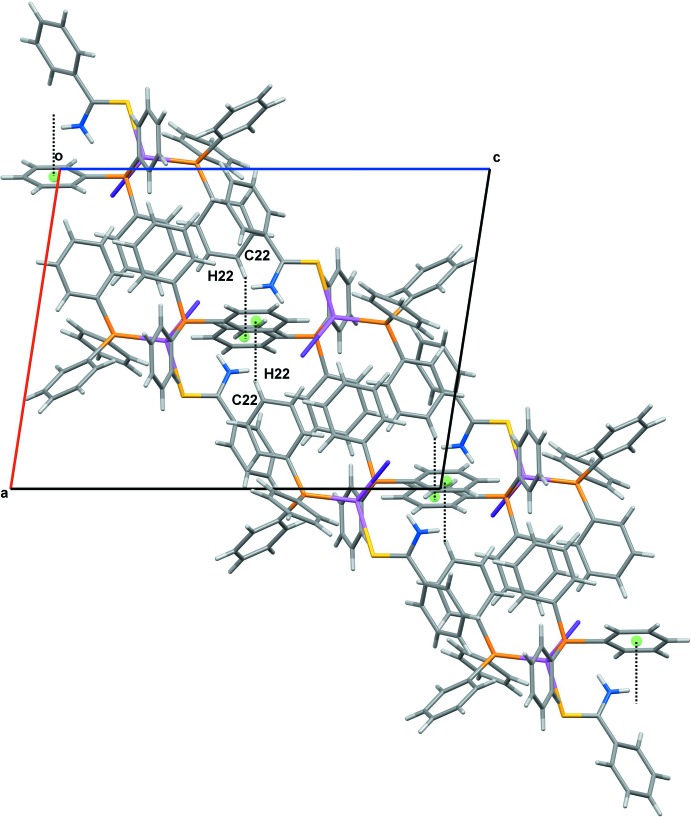
The supra­molecular C—H⋯π chain in the title compound.

In the crystal, the dimeric inter­molecular inter­actions are generated through a crystallographic inversion center by linking through the N1—H1*A*⋯Br1^i^ [3.357 (3)Å] and C17—H17⋯Br1^i^ [3.789 (3) Å] [symmetry code: (i) 1 – *x*, 1 – *y*, 1 – *z*] hydrogen bonds between a pair of adjacent complex mol­ecules; these are similar to the those in the above-mentioned complex [AgCl(C_7_H_7_NS)(C_18_H_15_P)_2_] (Ruangwut & Pakawatchai, 2014 ▸)*.* There are two cyclic patterns of 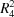
(8) loops formed by two pairs of N1—H1*A*⋯Br1 and N1—H1*B*⋯Br1 inter­actions and of 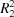
(14) loops forming by a pair of C17—H17⋯Br1 inter­actions, as illustrated in Fig. 2 ▸. In addition, supra­molecular C—H⋯π chains (Fig. 3 ▸) are formed between the C*sp* ^2^ atoms of the phenyl rings and the centroids of another phenyl ring [C22—H22⋯*Cg*3 = 3.782 (3) Å].

## Hirshfeld surface analysis   

**Figure 4:**
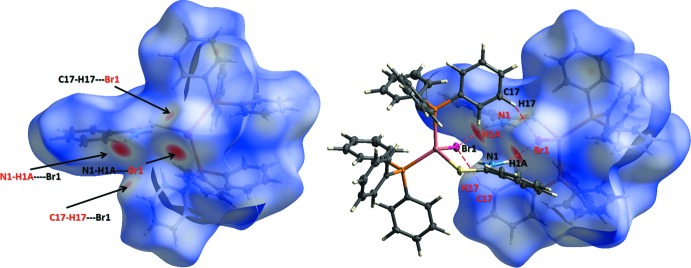
*d*_norm_ mapped on the Hirshfeld surface for visualizing the inter­molecular inter­actions of the title compound. Dotted red lines represent hydrogen bonds.

**Figure 5:**
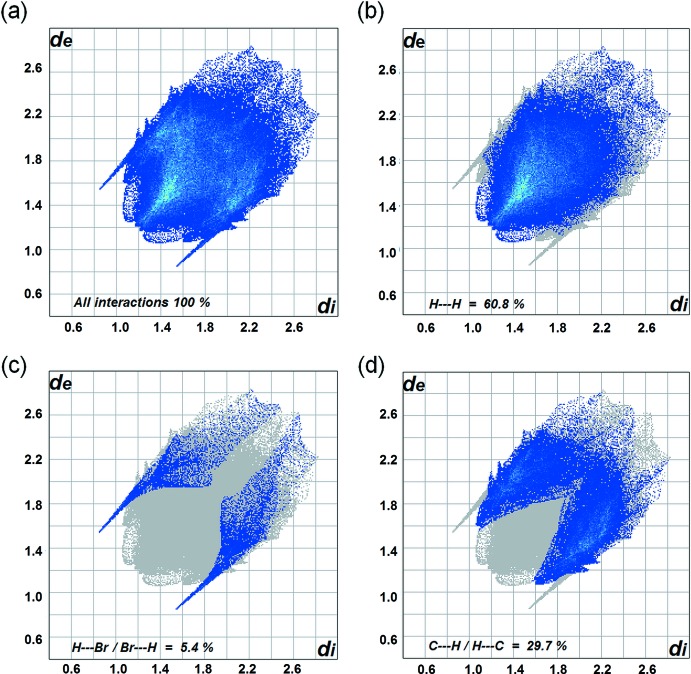
Two-dimensional fingerprint plots of the title complex showing the percentage contributions of individual types of inter­actions: (*a*) all inter­molecular inter­actions, (*b*) H⋯H contacts, (*c*) H⋯Br/Br⋯H contacts and (*d*) C⋯H/H⋯C contacts. *d*_e_ and *d*_i_ represent the distances from the surface to nearest external and inter­nal atoms and the blue–cyan color represents increasing numbers of surface contributors at individual *d*_e_/*d*_i_ points

For the title complex, the Hirshfeld-surfaces analysis (McKinnon *et al.*, 2004 ▸; Spackman & Jayatilaka, 2009 ▸) was generated by *Crystal Explorer 3.1* (Wolff *et al.*, 2012 ▸) and mapped over *d_norm_*, *d*_e_ and *d*_i_ fingerprint plot (Spackman & McKinnon 2002 ▸; McKinnon *et al.*, 2007 ▸). The contact distances to the closest atom inside (*d*_i_) and outside (*d*_e_) of the Hirshfeld surface analyse the inter­molecular inter­actions *via* the mapping of *d_norm_*, as depicted in Fig. 4 ▸. The inter­actions are shown on the Hirshfeld surfaces with short contacts indicated in red. The corresponding fingerprint plots (Fig. 5 ▸ *a*–*d*) for Hirshfeld surfaces of the complex are shown with characteristic pseudo-symmetry wings in the upper left and lower right sides of the *d*e and *d*i diagonal axes that represent the overall 2D fingerprint plot and those delineated into H⋯H, H⋯Br/Br⋯H, and C⋯H/H⋯C contacts are shown in Fig. 5 ▸ *a*–*d*, respectively. The fingerprint plot of H⋯H contacts represented by the largest contribution within the Hirshfeld surfaces (60.8%) are shown as one distinct pattern with a minimum value of *d*_e_ + *d_i_* ∼2.6 Å. The reciprocal H⋯Br/Br⋯H contacts consist of 5.4% of the total Hirshfeld surface with *d*_e_ + *d_i_* ∼3.3 Å, exhibited by two symmetrical narrow pointed wings indicating the inter­molecular hydrogen-bond inter­actions N1—H1*A*⋯Br1 and C17—H17⋯Br1 in the crystal packing. The presence of C—H⋯π inter­actions on the fingerprint plot, which contribute 29.7% of overall Hirshfeld surface, are indicated by *d*_e_ + *d_i_* ∼3.0 Å.

## Synthesis and crystallization   

Silver(I) bromide (0.10 g, 0.5 mmol) was dissolved in the mixed solvent of 15 ml of aceto­nitrile and 15 ml of ethanol and then tri­phenyl­phosphane (0.27 g, 1 mmol) was added. The mixture was refluxed for 2 h at 343 K and a white precipitate was formed. After that, benzene­carbo­thio­amide (0.13 g, 1 mmol) was added and continually refluxed for 5 h. At that time, the white precipitate dissolved. The clear yellow solution was filtered and left to evaporate at room temperature. After a day, pale-yellow blocks of the title compound were filtered off and dried *in vacuo*. Calculated for C_43_H_37_AgBrNP_2_S: C 61.07, H 4.37, N 1.65 and S 3.78%. Found: C 60.50, H 4.21, N 1.43 and S 3.70%.

## Refinement   

Crystal data and details of structure determination are summarized in Table 2 ▸. All H atoms on carbon atoms were positioned geometrically and refined using a riding-model approximation with C—H = 0.93 Å with *U*_iso_(H) = 1.2 *U*_eq_(C). N-bound H atoms were found from difference maps and refined isotropically with distance restraint N—H = 0.85–0.86 Å.

## Supplementary Material

Crystal structure: contains datablock(s) I. DOI: 10.1107/S2056989016009518/hb7592sup1.cif

Structure factors: contains datablock(s) I. DOI: 10.1107/S2056989016009518/hb7592Isup2.hkl

CCDC reference: 1484796

Additional supporting information: crystallographic information; 3D view; checkCIF report

## Figures and Tables

**Table 1:** Hydrogen-bond geometry (Å, °) *Cg*3 is the centroid of the C13–C18 ring.

*D*—H⋯*A*	*D*—H	H⋯*A*	*D*⋯*A*	*D*—H⋯*A*
N1—H1*A*⋯Br1^i^	0.85 (1)	2.54 (1)	3.357 (3)	161 (3)
N1—H1*B*⋯Br1	0.85 (1)	2.58 (1)	3.413 (3)	166 (3)
C17—H17⋯Br1^i^	0.93	2.91	3.789 (3)	158
C22—H22⋯*Cg*3^ii^	0.93	2.94	3.78 (3)	151

Symmetry codes: (i) 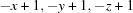; (ii) 
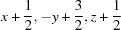
.

**Table 2:** Experimental details

Crystal data	
Chemical formula	[AgBr(C_7_H_7_NS)(C_18_H_15_P)_2_]
*M*_r_	849.51
Crystal system, space group	Monoclinic, *P*2_1_/*n*
Temperature (K)	293
*a*, *b*, *c* (Å)	14.4354 (5), 14.1925 (5), 19.1682 (6)
β (°)	98.786 (1)
*V* (Å^3^)	3881.0 (2)
*Z*	4
Radiation type	Mo *K*α
μ (mm^−1^)	1.72
Crystal size (mm)	0.23 × 0.13 × 0.08

Data collection	
Diffractometer	Bruker *APEX* CCD area-detector
Absorption correction	Multi-scan (*SADABS*; Bruker, 2003 ▸)
*T*_min_, *T*_max_	0.885, 1.000
No. of measured, independent and observed [*I* > 2σ(*I*)] reflections	52150, 9261, 6704
*R*_int_	0.048
(sin θ/λ)_max_ (Å^−1^)	0.658

Refinement	
*R*[*F* ^2^ > 2σ(*F* ^2^)], *wR*(*F* ^2^), *S*	0.036, 0.085, 1.02
No. of reflections	9261
No. of parameters	450
No. of restraints	2
H-atom treatment	H atoms treated by a mixture of independent and constrained refinement
Δρ_max_, Δρ_min_ (e Å^−3^)	0.47, −0.24

Computer programs: *SMART* and *SAINT* (Bruker, 2003 ▸), *SHELXT2014* (Sheldrick, 2015*a* ▸), *SHELXL2014* (Sheldrick, 2015*b* ▸), *Mercury* (Macrae *et al.*, 2008 ▸), *WinGX* (Farrugia, 2012 ▸) and *publCIF* (Westrip, 2010 ▸).

## supplementary crystallographic information

### Crystal data

**Table:**

[AgBr(C_7_H_7_NS)(C_18_H_15_P)_2_]	*F*(000) = 1720
*M**_r_* = 849.51	*D*_x_ = 1.454 Mg m^−^^3^
Monoclinic, *P*2_1_/*n*	Mo *K*α radiation, λ = 0.71073 Å
*a* = 14.4354 (5) Å	Cell parameters from 7218 reflections
*b* = 14.1925 (5) Å	θ = 2.4–22.5°
*c* = 19.1682 (6) Å	µ = 1.72 mm^−^^1^
β = 98.786 (1)°	*T* = 293 K
*V* = 3881.0 (2) Å^3^	Block, pale yellow
*Z* = 4	0.23 × 0.13 × 0.08 mm

### Data collection

**Table:**

Bruker APEX CCD area-detector diffractometer	6704 reflections with *I* > 2σ(*I*)
Radiation source: fine-focus sealed tube	*R*_int_ = 0.048
Frames, each covering 0.3 ° in ω scans	θ_max_ = 27.9°, θ_min_ = 1.7°
Absorption correction: multi-scan (SADABS; Bruker, 2003)	*h* = −18→18
*T*_min_ = 0.885, *T*_max_ = 1.000	*k* = −18→18
52150 measured reflections	*l* = −25→25
9261 independent reflections	

### Refinement

**Table:**

Refinement on *F*^2^	Primary atom site location: structure-invariant direct methods
Least-squares matrix: full	Secondary atom site location: difference Fourier map
*R*[*F*^2^ > 2σ(*F*^2^)] = 0.036	Hydrogen site location: mixed
*wR*(*F*^2^) = 0.085	H atoms treated by a mixture of independent and constrained refinement
*S* = 1.02	*w* = 1/[σ^2^(*F*_o_^2^) + (0.0406*P*)^2^ + 0.3053*P*] where *P* = (*F*_o_^2^ + 2*F*_c_^2^)/3
9261 reflections	(Δ/σ)_max_ = 0.002
450 parameters	Δρ_max_ = 0.47 e Å^−^^3^
2 restraints	Δρ_min_ = −0.24 e Å^−^^3^

### Special details

**Table:**

Geometry. All esds (except the esd in the dihedral angle between two l.s. planes) are estimated using the full covariance matrix. The cell esds are taken into account individually in the estimation of esds in distances, angles and torsion angles; correlations between esds in cell parameters are only used when they are defined by crystal symmetry. An approximate (isotropic) treatment of cell esds is used for estimating esds involving l.s. planes.

### Fractional atomic coordinates and isotropic or equivalent isotropic displacement parameters (Å^2^)

**Table:**

	*x*	*y*	*z*	*U*_iso_*/*U*_eq_	
Ag1	0.46287 (2)	0.62099 (2)	0.69031 (2)	0.04396 (7)	
Br1	0.58251 (2)	0.50868 (2)	0.63144 (2)	0.05659 (9)	
P2	0.48182 (4)	0.57124 (5)	0.81500 (3)	0.03691 (15)	
P1	0.52341 (5)	0.77367 (5)	0.65520 (3)	0.03869 (15)	
S1	0.29135 (5)	0.58599 (6)	0.63246 (4)	0.0582 (2)	
N1	0.36464 (19)	0.50017 (19)	0.53303 (13)	0.0570 (6)	
C31	0.46028 (17)	0.44578 (18)	0.82667 (13)	0.0400 (6)	
C13	0.52381 (17)	0.79219 (17)	0.56084 (13)	0.0400 (6)	
C19	0.59722 (17)	0.58766 (17)	0.86800 (13)	0.0398 (6)	
C25	0.40173 (17)	0.63235 (18)	0.86506 (13)	0.0418 (6)	
C1	0.64521 (18)	0.78817 (18)	0.69490 (14)	0.0446 (6)	
C7	0.46305 (18)	0.87882 (18)	0.68001 (13)	0.0431 (6)	
C38	0.20344 (19)	0.5373 (2)	0.49980 (13)	0.0479 (6)	
C8	0.5067 (2)	0.95988 (19)	0.70832 (14)	0.0496 (7)	
H8	0.5717	0.9634	0.7165	0.059*	
C14	0.5523 (2)	0.87597 (18)	0.53392 (15)	0.0505 (7)	
H14	0.5707	0.9259	0.5643	0.061*	
C37	0.29051 (18)	0.53868 (19)	0.55164 (13)	0.0438 (6)	
C26	0.3653 (2)	0.5943 (2)	0.92112 (15)	0.0575 (7)	
H26	0.3812	0.5330	0.9354	0.069*	
C18	0.4949 (2)	0.7197 (2)	0.51477 (14)	0.0567 (8)	
H18	0.4745	0.6633	0.5319	0.068*	
C36	0.4922 (2)	0.39729 (19)	0.88842 (15)	0.0527 (7)	
H36	0.5274	0.4286	0.9261	0.063*	
C15	0.5538 (2)	0.8863 (2)	0.46293 (16)	0.0570 (8)	
H15	0.5739	0.9427	0.4456	0.068*	
C32	0.4108 (2)	0.39653 (19)	0.77074 (15)	0.0533 (7)	
H32	0.3904	0.4272	0.7283	0.064*	
C9	0.4535 (3)	1.0364 (2)	0.72469 (15)	0.0627 (8)	
H9	0.4832	1.0904	0.7444	0.075*	
C30	0.3763 (2)	0.7235 (2)	0.84460 (17)	0.0589 (8)	
H30	0.3992	0.7501	0.8063	0.071*	
C6	0.7174 (2)	0.7775 (2)	0.65652 (18)	0.0662 (8)	
H6	0.7044	0.7668	0.6081	0.079*	
C35	0.4722 (2)	0.3025 (2)	0.89437 (16)	0.0629 (8)	
H35	0.4934	0.2708	0.9362	0.075*	
C20	0.6114 (2)	0.6257 (2)	0.93455 (15)	0.0625 (8)	
H20	0.5601	0.6456	0.9547	0.075*	
C16	0.5258 (2)	0.8140 (2)	0.41780 (15)	0.0638 (8)	
H16	0.5267	0.8212	0.3697	0.077*	
C34	0.4216 (2)	0.2551 (2)	0.83930 (18)	0.0647 (8)	
H34	0.4078	0.1916	0.8437	0.078*	
C12	0.3662 (2)	0.8761 (2)	0.66785 (19)	0.0675 (9)	
H12	0.3354	0.8221	0.6490	0.081*	
C22	0.7760 (2)	0.6065 (2)	0.9435 (2)	0.0730 (10)	
H22	0.8361	0.6122	0.9689	0.088*	
C17	0.4962 (3)	0.7304 (2)	0.44316 (16)	0.0703 (9)	
H17	0.4772	0.6812	0.4123	0.084*	
C33	0.3916 (2)	0.3018 (2)	0.77778 (18)	0.0676 (9)	
H33	0.3577	0.2695	0.7400	0.081*	
C43	0.1355 (2)	0.6041 (3)	0.50190 (18)	0.0769 (11)	
H43	0.1446	0.6509	0.5363	0.092*	
C27	0.3056 (2)	0.6455 (3)	0.95658 (19)	0.0755 (10)	
H27	0.2818	0.6189	0.9945	0.091*	
C24	0.6743 (2)	0.5599 (2)	0.83923 (16)	0.0646 (8)	
H24	0.6667	0.5344	0.7940	0.077*	
C28	0.2820 (2)	0.7350 (3)	0.9356 (2)	0.0835 (11)	
H28	0.2413	0.7693	0.9592	0.100*	
C39	0.1878 (2)	0.4692 (2)	0.44791 (17)	0.0699 (9)	
H39	0.2329	0.4232	0.4450	0.084*	
C2	0.6671 (2)	0.8013 (2)	0.76686 (15)	0.0621 (8)	
H2	0.6190	0.8066	0.7939	0.075*	
C3	0.7590 (3)	0.8066 (3)	0.7994 (2)	0.0811 (11)	
H3	0.7727	0.8166	0.8478	0.097*	
C29	0.3173 (2)	0.7755 (3)	0.8803 (2)	0.0775 (10)	
H29	0.3019	0.8372	0.8669	0.093*	
C23	0.7633 (2)	0.5695 (3)	0.8772 (2)	0.0807 (11)	
H23	0.8151	0.5505	0.8573	0.097*	
C21	0.7004 (2)	0.6350 (3)	0.97230 (18)	0.0788 (10)	
H21	0.7085	0.6608	1.0174	0.095*	
C4	0.8294 (3)	0.7974 (3)	0.7605 (2)	0.0887 (12)	
H4	0.8914	0.8012	0.7823	0.106*	
C10	0.3588 (3)	1.0325 (3)	0.71206 (19)	0.0775 (11)	
H10	0.3236	1.0839	0.7228	0.093*	
C41	0.0393 (3)	0.5356 (3)	0.40405 (18)	0.0859 (12)	
H41	−0.0162	0.5347	0.3722	0.103*	
C11	0.3150 (3)	0.9533 (3)	0.6836 (2)	0.0874 (12)	
H11	0.2499	0.9512	0.6746	0.105*	
C5	0.8099 (2)	0.7825 (3)	0.6898 (2)	0.0874 (11)	
H5	0.8586	0.7757	0.6636	0.105*	
C40	0.1056 (3)	0.4688 (3)	0.4003 (2)	0.0889 (12)	
H40	0.0957	0.4226	0.3656	0.107*	
C42	0.0537 (3)	0.6035 (3)	0.4541 (2)	0.0978 (14)	
H42	0.0085	0.6497	0.4562	0.117*	
H1A	0.370 (2)	0.485 (2)	0.4908 (7)	0.064 (10)*	
H1B	0.4142 (14)	0.498 (2)	0.5637 (13)	0.071 (10)*	

### Atomic displacement parameters (Å^2^)

**Table:**

	*U*^11^	*U*^22^	*U*^33^	*U*^12^	*U*^13^	*U*^23^
Ag1	0.05487 (13)	0.04402 (12)	0.03328 (10)	−0.00277 (9)	0.00765 (8)	0.00067 (8)
Br1	0.06087 (19)	0.06224 (19)	0.04602 (16)	0.01813 (14)	0.00608 (13)	−0.00806 (13)
P2	0.0384 (4)	0.0428 (4)	0.0294 (3)	−0.0038 (3)	0.0046 (3)	−0.0006 (3)
P1	0.0405 (4)	0.0387 (4)	0.0364 (3)	−0.0011 (3)	0.0043 (3)	0.0022 (3)
S1	0.0462 (4)	0.0899 (6)	0.0381 (4)	0.0004 (4)	0.0049 (3)	−0.0146 (4)
N1	0.0511 (16)	0.0765 (18)	0.0416 (15)	0.0049 (13)	0.0016 (12)	−0.0149 (13)
C31	0.0390 (14)	0.0432 (14)	0.0395 (14)	−0.0028 (11)	0.0117 (11)	−0.0001 (11)
C13	0.0413 (14)	0.0394 (14)	0.0392 (13)	0.0008 (11)	0.0056 (11)	0.0026 (11)
C19	0.0410 (14)	0.0418 (13)	0.0357 (13)	−0.0033 (11)	0.0032 (11)	0.0038 (11)
C25	0.0335 (13)	0.0524 (16)	0.0381 (13)	−0.0018 (11)	0.0009 (10)	−0.0091 (11)
C1	0.0417 (15)	0.0399 (14)	0.0508 (16)	−0.0016 (11)	0.0031 (12)	0.0019 (12)
C7	0.0475 (15)	0.0438 (15)	0.0387 (14)	0.0029 (12)	0.0090 (11)	0.0079 (11)
C38	0.0470 (16)	0.0564 (17)	0.0388 (15)	−0.0034 (13)	0.0017 (12)	−0.0017 (12)
C8	0.0599 (18)	0.0475 (16)	0.0412 (15)	0.0022 (14)	0.0076 (13)	0.0020 (12)
C14	0.0604 (18)	0.0443 (15)	0.0478 (16)	−0.0077 (13)	0.0111 (13)	−0.0001 (12)
C37	0.0480 (16)	0.0474 (15)	0.0358 (13)	−0.0048 (12)	0.0062 (11)	−0.0004 (11)
C26	0.0495 (17)	0.076 (2)	0.0486 (17)	0.0003 (15)	0.0137 (13)	−0.0011 (15)
C18	0.081 (2)	0.0434 (16)	0.0426 (16)	−0.0081 (14)	−0.0016 (14)	0.0039 (12)
C36	0.0664 (19)	0.0514 (17)	0.0409 (15)	−0.0057 (14)	0.0105 (13)	0.0014 (12)
C15	0.069 (2)	0.0506 (17)	0.0537 (18)	−0.0045 (14)	0.0185 (15)	0.0128 (14)
C32	0.0589 (18)	0.0466 (17)	0.0518 (17)	−0.0013 (13)	−0.0006 (14)	−0.0021 (13)
C9	0.099 (3)	0.0502 (18)	0.0401 (16)	0.0130 (17)	0.0152 (16)	0.0038 (13)
C30	0.0547 (18)	0.0578 (19)	0.0644 (19)	0.0051 (14)	0.0103 (15)	−0.0063 (15)
C6	0.0484 (18)	0.083 (2)	0.067 (2)	0.0052 (16)	0.0095 (15)	−0.0037 (17)
C35	0.086 (2)	0.0514 (18)	0.0558 (18)	0.0008 (16)	0.0264 (17)	0.0143 (15)
C20	0.0428 (16)	0.094 (2)	0.0492 (17)	−0.0026 (16)	0.0020 (13)	−0.0187 (16)
C16	0.088 (2)	0.067 (2)	0.0371 (15)	0.0064 (18)	0.0116 (15)	0.0091 (15)
C34	0.075 (2)	0.0433 (17)	0.080 (2)	−0.0069 (15)	0.0283 (18)	−0.0005 (16)
C12	0.0484 (18)	0.0524 (18)	0.102 (3)	0.0035 (15)	0.0115 (17)	0.0094 (17)
C22	0.0412 (18)	0.090 (3)	0.081 (3)	−0.0003 (17)	−0.0120 (16)	0.005 (2)
C17	0.112 (3)	0.0534 (19)	0.0414 (16)	−0.0049 (18)	−0.0030 (17)	−0.0042 (14)
C33	0.075 (2)	0.0524 (19)	0.072 (2)	−0.0125 (16)	0.0024 (17)	−0.0101 (16)
C43	0.074 (2)	0.093 (3)	0.057 (2)	0.022 (2)	−0.0118 (17)	−0.0258 (18)
C27	0.055 (2)	0.107 (3)	0.069 (2)	−0.001 (2)	0.0270 (17)	−0.015 (2)
C24	0.0506 (18)	0.085 (2)	0.0585 (19)	0.0097 (17)	0.0088 (15)	−0.0137 (17)
C28	0.057 (2)	0.107 (3)	0.088 (3)	0.007 (2)	0.019 (2)	−0.039 (2)
C39	0.068 (2)	0.071 (2)	0.066 (2)	0.0066 (17)	−0.0053 (16)	−0.0185 (17)
C2	0.0574 (19)	0.073 (2)	0.0520 (18)	−0.0008 (16)	−0.0031 (14)	0.0007 (15)
C3	0.076 (3)	0.085 (3)	0.072 (2)	−0.008 (2)	−0.022 (2)	−0.0021 (19)
C29	0.065 (2)	0.068 (2)	0.097 (3)	0.0170 (18)	0.006 (2)	−0.018 (2)
C23	0.0428 (18)	0.110 (3)	0.089 (3)	0.0174 (19)	0.0091 (17)	−0.007 (2)
C21	0.059 (2)	0.111 (3)	0.060 (2)	−0.005 (2)	−0.0114 (16)	−0.0200 (19)
C4	0.051 (2)	0.091 (3)	0.115 (4)	−0.007 (2)	−0.019 (2)	0.002 (2)
C10	0.101 (3)	0.065 (2)	0.076 (2)	0.036 (2)	0.040 (2)	0.0163 (18)
C41	0.064 (2)	0.128 (3)	0.057 (2)	0.006 (2)	−0.0167 (17)	−0.015 (2)
C11	0.058 (2)	0.082 (3)	0.128 (3)	0.022 (2)	0.031 (2)	0.020 (2)
C5	0.045 (2)	0.108 (3)	0.111 (3)	0.0058 (19)	0.017 (2)	0.005 (3)
C40	0.083 (3)	0.101 (3)	0.072 (2)	0.000 (2)	−0.020 (2)	−0.035 (2)
C42	0.082 (3)	0.130 (4)	0.071 (2)	0.043 (2)	−0.021 (2)	−0.027 (2)

### Geometric parameters (Å, º)

**Table:**

Ag1—P2	2.4671 (6)	C30—H30	0.9300
Ag1—P1	2.4682 (7)	C6—C5	1.391 (5)
Ag1—S1	2.6015 (8)	C6—H6	0.9300
Ag1—Br1	2.7189 (3)	C35—C34	1.365 (4)
P2—C31	1.827 (3)	C35—H35	0.9300
P2—C19	1.829 (2)	C20—C21	1.381 (4)
P2—C25	1.830 (3)	C20—H20	0.9300
P1—C1	1.818 (3)	C16—C17	1.374 (4)
P1—C7	1.827 (3)	C16—H16	0.9300
P1—C13	1.828 (2)	C34—C33	1.364 (4)
S1—C37	1.687 (3)	C34—H34	0.9300
N1—C37	1.299 (4)	C12—C11	1.380 (5)
N1—H1A	0.852 (10)	C12—H12	0.9300
N1—H1B	0.854 (10)	C22—C21	1.358 (5)
C31—C32	1.383 (3)	C22—C23	1.361 (5)
C31—C36	1.386 (3)	C22—H22	0.9300
C13—C18	1.378 (4)	C17—H17	0.9300
C13—C14	1.384 (3)	C33—H33	0.9300
C19—C20	1.371 (4)	C43—C42	1.380 (5)
C19—C24	1.373 (4)	C43—H43	0.9300
C25—C26	1.377 (4)	C27—C28	1.361 (5)
C25—C30	1.385 (4)	C27—H27	0.9300
C1—C6	1.373 (4)	C24—C23	1.383 (4)
C1—C2	1.380 (4)	C24—H24	0.9300
C7—C8	1.382 (4)	C28—C29	1.372 (5)
C7—C12	1.383 (4)	C28—H28	0.9300
C38—C43	1.370 (4)	C39—C40	1.382 (4)
C38—C39	1.381 (4)	C39—H39	0.9300
C38—C37	1.479 (4)	C2—C3	1.380 (4)
C8—C9	1.393 (4)	C2—H2	0.9300
C8—H8	0.9300	C3—C4	1.355 (5)
C14—C15	1.372 (4)	C3—H3	0.9300
C14—H14	0.9300	C29—H29	0.9300
C26—C27	1.383 (4)	C23—H23	0.9300
C26—H26	0.9300	C21—H21	0.9300
C18—C17	1.384 (4)	C4—C5	1.357 (5)
C18—H18	0.9300	C4—H4	0.9300
C36—C35	1.385 (4)	C10—C11	1.363 (5)
C36—H36	0.9300	C10—H10	0.9300
C15—C16	1.363 (4)	C41—C42	1.353 (5)
C15—H15	0.9300	C41—C40	1.357 (5)
C32—C33	1.384 (4)	C41—H41	0.9300
C32—H32	0.9300	C11—H11	0.9300
C9—C10	1.352 (5)	C5—H5	0.9300
C9—H9	0.9300	C40—H40	0.9300
C30—C29	1.384 (4)	C42—H42	0.9300
			
P2—Ag1—P1	121.60 (2)	C34—C35—C36	120.6 (3)
P2—Ag1—S1	108.40 (2)	C34—C35—H35	119.7
P1—Ag1—S1	113.89 (3)	C36—C35—H35	119.7
P2—Ag1—Br1	104.528 (18)	C19—C20—C21	121.3 (3)
P1—Ag1—Br1	97.338 (18)	C19—C20—H20	119.3
S1—Ag1—Br1	109.550 (19)	C21—C20—H20	119.3
C31—P2—C19	102.34 (11)	C15—C16—C17	120.2 (3)
C31—P2—C25	105.36 (12)	C15—C16—H16	119.9
C19—P2—C25	104.08 (11)	C17—C16—H16	119.9
C31—P2—Ag1	113.69 (8)	C33—C34—C35	119.4 (3)
C19—P2—Ag1	117.29 (8)	C33—C34—H34	120.3
C25—P2—Ag1	112.76 (9)	C35—C34—H34	120.3
C1—P1—C7	105.53 (12)	C11—C12—C7	120.1 (3)
C1—P1—C13	104.41 (12)	C11—C12—H12	119.9
C7—P1—C13	102.34 (11)	C7—C12—H12	119.9
C1—P1—Ag1	110.20 (8)	C21—C22—C23	119.5 (3)
C7—P1—Ag1	116.31 (8)	C21—C22—H22	120.3
C13—P1—Ag1	116.78 (8)	C23—C22—H22	120.3
C37—S1—Ag1	109.90 (10)	C16—C17—C18	119.9 (3)
C37—N1—H1A	124 (2)	C16—C17—H17	120.1
C37—N1—H1B	118 (2)	C18—C17—H17	120.1
H1A—N1—H1B	117 (3)	C34—C33—C32	120.9 (3)
C32—C31—C36	118.4 (2)	C34—C33—H33	119.5
C32—C31—P2	118.3 (2)	C32—C33—H33	119.5
C36—C31—P2	123.2 (2)	C38—C43—C42	121.3 (3)
C18—C13—C14	118.7 (2)	C38—C43—H43	119.3
C18—C13—P1	118.69 (19)	C42—C43—H43	119.3
C14—C13—P1	122.6 (2)	C28—C27—C26	119.5 (3)
C20—C19—C24	118.1 (2)	C28—C27—H27	120.2
C20—C19—P2	124.1 (2)	C26—C27—H27	120.2
C24—C19—P2	117.8 (2)	C19—C24—C23	120.3 (3)
C26—C25—C30	118.1 (3)	C19—C24—H24	119.9
C26—C25—P2	124.8 (2)	C23—C24—H24	119.9
C30—C25—P2	117.1 (2)	C27—C28—C29	120.9 (3)
C6—C1—C2	118.3 (3)	C27—C28—H28	119.6
C6—C1—P1	121.9 (2)	C29—C28—H28	119.6
C2—C1—P1	119.5 (2)	C38—C39—C40	120.5 (3)
C8—C7—C12	118.5 (3)	C38—C39—H39	119.8
C8—C7—P1	125.1 (2)	C40—C39—H39	119.8
C12—C7—P1	116.3 (2)	C1—C2—C3	121.1 (3)
C43—C38—C39	117.8 (3)	C1—C2—H2	119.5
C43—C38—C37	120.7 (3)	C3—C2—H2	119.5
C39—C38—C37	121.5 (3)	C4—C3—C2	119.8 (4)
C7—C8—C9	120.2 (3)	C4—C3—H3	120.1
C7—C8—H8	119.9	C2—C3—H3	120.1
C9—C8—H8	119.9	C28—C29—C30	119.2 (3)
C15—C14—C13	120.8 (3)	C28—C29—H29	120.4
C15—C14—H14	119.6	C30—C29—H29	120.4
C13—C14—H14	119.6	C22—C23—C24	120.8 (3)
N1—C37—C38	117.5 (2)	C22—C23—H23	119.6
N1—C37—S1	121.9 (2)	C24—C23—H23	119.6
C38—C37—S1	120.6 (2)	C22—C21—C20	120.0 (3)
C25—C26—C27	121.2 (3)	C22—C21—H21	120.0
C25—C26—H26	119.4	C20—C21—H21	120.0
C27—C26—H26	119.4	C3—C4—C5	120.4 (3)
C13—C18—C17	120.3 (3)	C3—C4—H4	119.8
C13—C18—H18	119.9	C5—C4—H4	119.8
C17—C18—H18	119.9	C9—C10—C11	120.0 (3)
C35—C36—C31	120.4 (3)	C9—C10—H10	120.0
C35—C36—H36	119.8	C11—C10—H10	120.0
C31—C36—H36	119.8	C42—C41—C40	120.1 (3)
C16—C15—C14	120.0 (3)	C42—C41—H41	120.0
C16—C15—H15	120.0	C40—C41—H41	120.0
C14—C15—H15	120.0	C10—C11—C12	120.8 (3)
C31—C32—C33	120.2 (3)	C10—C11—H11	119.6
C31—C32—H32	119.9	C12—C11—H11	119.6
C33—C32—H32	119.9	C4—C5—C6	120.3 (4)
C10—C9—C8	120.3 (3)	C4—C5—H5	119.9
C10—C9—H9	119.8	C6—C5—H5	119.9
C8—C9—H9	119.8	C41—C40—C39	120.3 (3)
C29—C30—C25	121.0 (3)	C41—C40—H40	119.8
C29—C30—H30	119.5	C39—C40—H40	119.8
C25—C30—H30	119.5	C41—C42—C43	119.9 (3)
C1—C6—C5	120.2 (3)	C41—C42—H42	120.0
C1—C6—H6	119.9	C43—C42—H42	120.0
C5—C6—H6	119.9		
			
C19—P2—C31—C32	−145.9 (2)	P1—C13—C18—C17	−178.6 (2)
C25—P2—C31—C32	105.5 (2)	C32—C31—C36—C35	−2.1 (4)
Ag1—P2—C31—C32	−18.4 (2)	P2—C31—C36—C35	178.9 (2)
C19—P2—C31—C36	33.1 (2)	C13—C14—C15—C16	0.8 (5)
C25—P2—C31—C36	−75.4 (2)	C36—C31—C32—C33	2.1 (4)
Ag1—P2—C31—C36	160.6 (2)	P2—C31—C32—C33	−178.9 (2)
C1—P1—C13—C18	119.0 (2)	C7—C8—C9—C10	−1.0 (4)
C7—P1—C13—C18	−131.2 (2)	C26—C25—C30—C29	1.2 (4)
Ag1—P1—C13—C18	−3.0 (3)	P2—C25—C30—C29	−178.8 (2)
C1—P1—C13—C14	−60.7 (2)	C2—C1—C6—C5	−1.8 (5)
C7—P1—C13—C14	49.1 (2)	P1—C1—C6—C5	−175.4 (3)
Ag1—P1—C13—C14	177.32 (19)	C31—C36—C35—C34	0.7 (5)
C31—P2—C19—C20	−101.0 (3)	C24—C19—C20—C21	−0.8 (5)
C25—P2—C19—C20	8.6 (3)	P2—C19—C20—C21	179.1 (3)
Ag1—P2—C19—C20	133.9 (2)	C14—C15—C16—C17	−0.2 (5)
C31—P2—C19—C24	78.9 (2)	C36—C35—C34—C33	0.6 (5)
C25—P2—C19—C24	−171.6 (2)	C8—C7—C12—C11	0.3 (5)
Ag1—P2—C19—C24	−46.3 (3)	P1—C7—C12—C11	−178.6 (3)
C31—P2—C25—C26	24.4 (3)	C15—C16—C17—C18	0.0 (5)
C19—P2—C25—C26	−82.9 (2)	C13—C18—C17—C16	−0.5 (5)
Ag1—P2—C25—C26	148.9 (2)	C35—C34—C33—C32	−0.7 (5)
C31—P2—C25—C30	−155.6 (2)	C31—C32—C33—C34	−0.7 (5)
C19—P2—C25—C30	97.1 (2)	C39—C38—C43—C42	0.3 (6)
Ag1—P2—C25—C30	−31.0 (2)	C37—C38—C43—C42	−179.7 (3)
C7—P1—C1—C6	−129.4 (2)	C25—C26—C27—C28	0.3 (5)
C13—P1—C1—C6	−21.9 (3)	C20—C19—C24—C23	0.6 (5)
Ag1—P1—C1—C6	104.2 (2)	P2—C19—C24—C23	−179.2 (3)
C7—P1—C1—C2	57.0 (3)	C26—C27—C28—C29	−0.7 (6)
C13—P1—C1—C2	164.5 (2)	C43—C38—C39—C40	−0.5 (5)
Ag1—P1—C1—C2	−69.4 (2)	C37—C38—C39—C40	179.5 (3)
C1—P1—C7—C8	13.5 (3)	C6—C1—C2—C3	2.1 (5)
C13—P1—C7—C8	−95.4 (2)	P1—C1—C2—C3	175.9 (3)
Ag1—P1—C7—C8	136.0 (2)	C1—C2—C3—C4	−1.1 (5)
C1—P1—C7—C12	−167.7 (2)	C27—C28—C29—C30	1.3 (5)
C13—P1—C7—C12	83.3 (2)	C25—C30—C29—C28	−1.6 (5)
Ag1—P1—C7—C12	−45.2 (2)	C21—C22—C23—C24	−0.6 (6)
C12—C7—C8—C9	0.6 (4)	C19—C24—C23—C22	0.1 (6)
P1—C7—C8—C9	179.4 (2)	C23—C22—C21—C20	0.5 (6)
C18—C13—C14—C15	−1.3 (4)	C19—C20—C21—C22	0.2 (6)
P1—C13—C14—C15	178.4 (2)	C2—C3—C4—C5	−0.3 (6)
C43—C38—C37—N1	−154.4 (3)	C8—C9—C10—C11	0.4 (5)
C39—C38—C37—N1	25.6 (4)	C9—C10—C11—C12	0.6 (6)
C43—C38—C37—S1	27.4 (4)	C7—C12—C11—C10	−0.9 (6)
C39—C38—C37—S1	−152.6 (3)	C3—C4—C5—C6	0.6 (6)
Ag1—S1—C37—N1	19.3 (3)	C1—C6—C5—C4	0.5 (6)
Ag1—S1—C37—C38	−162.44 (19)	C42—C41—C40—C39	0.6 (7)
C30—C25—C26—C27	−0.5 (4)	C38—C39—C40—C41	0.1 (6)
P2—C25—C26—C27	179.5 (2)	C40—C41—C42—C43	−0.8 (7)
C14—C13—C18—C17	1.2 (4)	C38—C43—C42—C41	0.4 (7)

### Hydrogen-bond geometry (Å, º)

Cg3 is the centroid of the C13–C18 ring.

**Table:**

*D*—H···*A*	*D*—H	H···*A*	*D*···*A*	*D*—H···*A*
N1—H1*A*···Br1^i^	0.85 (1)	2.54 (1)	3.357 (3)	161 (3)
N1—H1*B*···Br1	0.85 (1)	2.58 (1)	3.413 (3)	166 (3)
C17—H17···Br1^i^	0.93	2.91	3.789 (3)	158
C22—H22···*Cg*3^ii^	0.93	2.94	3.78 (3)	151

Symmetry codes: (i) −*x*+1, −*y*+1, −*z*+1; (ii) *x*+1/2, −*y*+3/2, *z*+1/2.
